# PKD phosphorylation and COP9/Signalosome modulate intracellular Spry2 protein stability

**DOI:** 10.1038/s41389-023-00465-3

**Published:** 2023-04-12

**Authors:** Natalia Martínez, Teresa Gragera, María Pilar de Lucas, Ana Belén Cámara, Alicia Ballester, Berta Anta, Alberto Fernández-Medarde, Tania López-Briones, Judith Ortega, Daniel Peña-Jiménez, Antonio Barbáchano, Ana Montero-Calle, Víctor Cordero, Rodrigo Barderas, Teresa Iglesias, Mónica Yunta, José Luís Oliva, Alberto Muñoz, Eugenio Santos, Natasha Zarich, José M. Rojas-Cabañeros

**Affiliations:** 1 Unidad Funcional de Investigación de Enfermedades Crónicas (UFIEC) and CIBERONC, Instituto de Salud Carlos III, 28220 Majadahonda, Madrid Spain; 2 Facultad de Odontología, Universidad Alfonso X el Sabio (UAX), Avenida de la Universidad 1, 28691 Villanueva de la Cañada, Madrid, Spain; 3 Centro de Investigación del Cáncer, IBMCC (CSIC-USAL) and Centro de Investigación Biomédica en Red de Cáncer (CIBERONC), Universidad de Salamanca, 37007 Salamanca, Spain; 4 Unidad de Investigación Biomédica, Universidad Alfonso X el Sabio (UAX), Avenida de la Universidad 1, 28691 Villanueva de la Cañada, Madrid, Spain; 5 Instituto de Investigaciones Biomédicas Alberto Sols and Centro de Investigación Biomédica en Red de Cáncer (CIBERONC), Consejo Superior de Investigaciones Científicas and Universidad Autónoma de Madrid (CSIC-UAM), 28029 Madrid, Spain; 6 Instituto de Investigación Sanitaria del Hospital Universitario La Paz (IdiPAZ), 28046 Madrid, Spain; 7 Instituto de Investigaciones Biomédicas Alberto Sols and Centro de Investigación Biomédica en Red de Enfermedades Neurodegenerativas (CIBERNED, Consejo Superior de Investigaciones Científicas and Universidad Autónoma de Madrid (CSIC-UAM), 28029 Madrid, Spain

**Keywords:** Checkpoint signalling, Targeted therapies, Ubiquitylation

## Abstract

Spry2 is a molecular modulator of tyrosine kinase receptor signaling pathways that has cancer-type-specific effects. Mammalian Spry2 protein undergoes tyrosine and serine phosphorylation in response to growth factor stimulation. Spry2 expression is distinctly altered in various cancer types. Inhibition of the proteasome functionality results in reduced intracellular Spry2 degradation. Using in vitro and in vivo assays, we show that protein kinase D (PKD) phosphorylates Spry2 at serine 112 and interacts in vivo with the C-terminal half of this protein. Importantly, missense mutation of Ser112 decreases the rate of Spry2 intracellular protein degradation. Either knocking down the expression of all three mammalian PKD isoforms or blocking their kinase activity with a specific inhibitor contributes to the stabilization of Spry2 wild-type protein. Downregulation of CSN3, a component of the COP9/Signalosome that binds PKD, significantly increases the half-life of Spry2 wild-type protein but does not affect the stability of a Spry2 after mutating Ser112 to the non-phosphorylatable residue alanine. Our data demonstrate that both PKD and the COP9/Signalosome play a significant role in control of Spry2 intracellular stability and support the consideration of the PKD/COP9 complex as a potential therapeutic target in tumors where Spry2 expression is reduced.

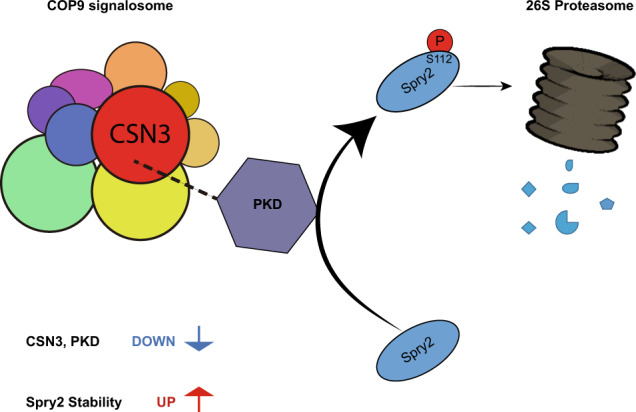

## Introduction

Sprouty (Spry) was initially identified in *Drosophila melanogaster* (dSpry) as a modulator of receptor tyrosine kinase (RTK) signaling during different morphogenetic processes [1]. Four mammalian genes (Spry1–4) that encode protein homologs of dSpry [2] have been identified, with Spry2 ubiquitously expressed in adult tissues [3]. Spry2 overexpression inhibits ERK activation by fibroblast growth factor (FGF) and vascular endothelial growth factor (VEGF), but not by epidermal growth factor (EGF) [1].

Consistent with these RTK-dependent activities of Spry2, its behavior in cancer shows opposite effects. Thus, we and others have proven that Spry2 displays tumor suppressive effects in breast, prostate, and liver cancers [4], as well as in B-cell diffuse lymphoma [5]. In contrast, in colorectal cancer (CRC), we have reported Spry2 upregulation in high-grade tumors and at the invasion front of low-grade tumors [6], where the levels of Spry2 RNA and protein are higher in colon adenocarcinomas than in the adjacent normal mucosa [7]. In addition, Spry2 triggers increased levels of c-MET and HGF-stimulated phosphorylation of ERK and AKT in HT-29 and LS-174T cells, promoting cell migration and invasion [7]. Moreover, Spry2 is upregulated in K-RAS mutant CRC [8] and in melanoma cells harboring N-RAS or B-RAF mutations [9]. In line with these observations, it has also been shown that Spry2 overexpression in fibroblasts, which provides sustained EGFR signaling, facilitates cell overgrowth and oncogenic transformation [10]. Mechanistically, this cellular behavior usually results from the molecular binding of Spry2 to the ubiquitin-ligase c-Cbl and the endocytic protein CIN85, involved in receptor endocytosis and degradation [11]. ITSN1 and p38^MAPK^ enhance the c-Cbl effects on EGFR, either by disrupting the interaction of Spry2 with c-Cbl [12], or by inducing Spry2 downregulation through activation of the E3 ubiquitin-ligase Siah2 [10], respectively.

Consistent with its role as a docking/scaffold protein, Spry2 does not have an enzymatic activity but its biological function is modulated by direct phosphorylation mediated by tyrosine- and serine/threonine kinases [1], at specific residues that are essential for the function and stability of this protein. Specifically, phosphorylation at Y55 affects FGF-signaling, whereas it has no effect on EGF-stimulated signaling [1]. In contrast, phosphorylation at T75 by the serine/threonine-kinase DYRK1A impairs the inhibitory activity of Spry2 on FGFR-mediated ERK signaling [13], and also prevents the endocytosis-mediated degradation of EGFR during asymmetric neural stem cell division [14]. Mnk1 also phosphorylates Spry2 (at S112 and S121 residues) increasing its stability [15], and is necessary for the capacity of Spry2 to interfere with intracellular trafficking of the activated EGFR at the step of progression from early to late endosomes [16].

Based on protein sequence analysis, herein we have found that the serine 112 of Spry2 constitutes a canonical protein kinase D (PKD) phosphorylation site. Furthermore, our subsequent in vitro and in vivo studies demonstrate that this residue is indeed phosphorylated by PKD. In addition, we show that inactivating PKD, or blocking the expression of CSN3, a subunit of the highly conserved COP9/Signalosome (CSN) complex [17] with binding affinity to PKD, leads to the stabilization of Spry2.

## Results

### PKD1 phosphorylates Spry2 at serine 112

**Fig. 1:**
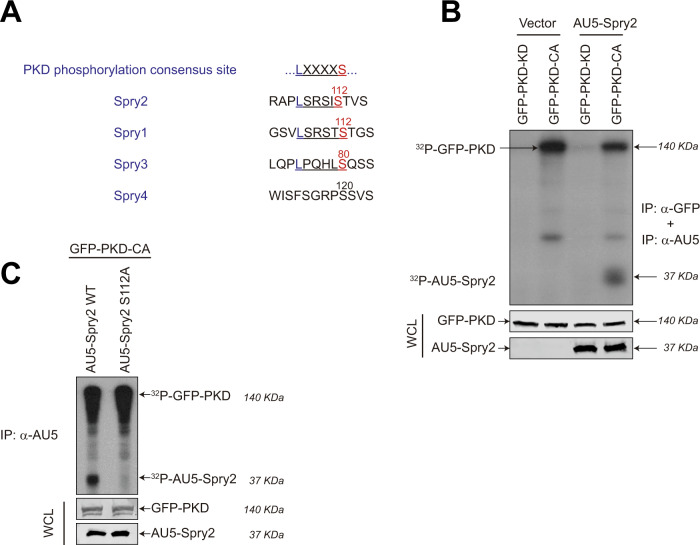
PKD phosphorylates Spry2 at serine 112 in vitro and in vivo. **A** PKD phosphorylation consensus site around serine 112 in Spry2 and Spry1, and serine 80 in Spry3. **B** PKD phosphorylates Spry2 in vitro. HEK293T cells were transfected with 5 µg of pECFL-AU5-Spry2 or AU5-vector, and with pEFBOS-GFP-PKD1-KD (GFP-PKD-KD) or pEFBOS-GFP-PKD1-CA (GFP-PKD-CA) for 48 h. Then, cells were serum-starved for 18 h, lysated and 200 µg of whole-cell lysates (WCL) were immunoprecipitated with anti-AU5 and anti-GFP antibodies. The expression levels of transfected proteins were detected by immunoblotting with specific antibodies after WCL were resolved by SDS-PAGE. Autoradiogram of in vitro kinase assays performed in the immunoprecipates using [γ^32^P]-ATP showing phosphorylated AU5-Spry2 and autophosphorylated GFP-PKD-CA. **C** PKD phosphorylates Spry2 in vivo at serine 112. HEK293T cells were transfected with 5 µg of AU5-Spry2 WT or AU5-Spry2 S112A together with pEFBOS-GFP-PKD1-CA (GFP-PKD-CA). Cells were in vivo labeled using ^32^P_i_ as described in Materials and Methods. AU5-immunoprecipates were resolved by SDS-PAGE. Radioactive bands in the autoradiogram correspond to AU5-Spry2 and autophosphorylated GFP-PKD-CA. Expression levels of transfected proteins were detected by immunoblotting with specific antibodies after WCL were resolved by SDS-PAGE.

Looking for kinases potentially able to act on Spry2, we observed that the serine at position 112 (S112) in this protein is part of an amino acid stretch that matches one of the three consensus phosphorylation sequences for PKD. The established PKD target motif (L/V/I)*X*(R/K)*XX*(S/T) [18] corresponds to the sequence “LSRSIS^112^” within Spry2 (Fig. 1A), and is also contained in Spry1 and Spry3, but not in Spry4 (Fig. 1A).

In order to determine whether PKD was able to phosphorylate Spry2, anti-AU5 immunoprecipitates obtained from lysates of HEK293T cells previously transfected with constructs coding for the full-length form of human Spry2 protein (AU5-Spry2) or AU5-vector (Vector) were used as substrates for in vitro kinase (IVK) assays with PKD1-KD (kinase dead) and PKD1-CA (constitutively active) mutants of PKD1 [19] (Fig. 1B). These two PKD1 mutant forms were expressed in HEK293T cells after transfection of pEFBOS-GFP-PKD1-CA (GFP-PKD-CA) and pEFBOS-GFP-PKD1-KD (GFP-PKD-KD) and immunoprecipitated with anti-GFP antibodies to be used in the in vitro kinase (IVK) assays. Results showed a clear phosphorylation of Spry2 by PKD1-CA but not by PKD1-KD (Fig. 1B). Consistent with previous reports [19], our assays also detected autophosphorylation of PKD1-CA (Fig. 1B).

Furthermore, in vivo phosphorylation assays with inorganic ^32^P demonstrated that PKD1-CA elicited the phosphorylation of WT AU5-Spry2 in living HEK293T cells co-transfected with vectors for the ectopic expression of both proteins, but it did not happen with a mutant counterpart AU5-Spry2 S112A, where serine 112 was switched to the non-phosphorylatable residue alanine (Fig. 1C). In these assays, we could also detect the presence of autophosphorylated PKD1-CA in AU5-Spry2 immunoprecipitates, indicative of the association of both molecules. Taken together, all these results support that PKD1 associates with and phosphorylates Spry2 specifically at serine 112.

### The C-terminal half of Spry2 mediates its association with PKD in vivo

**Fig. 2:**
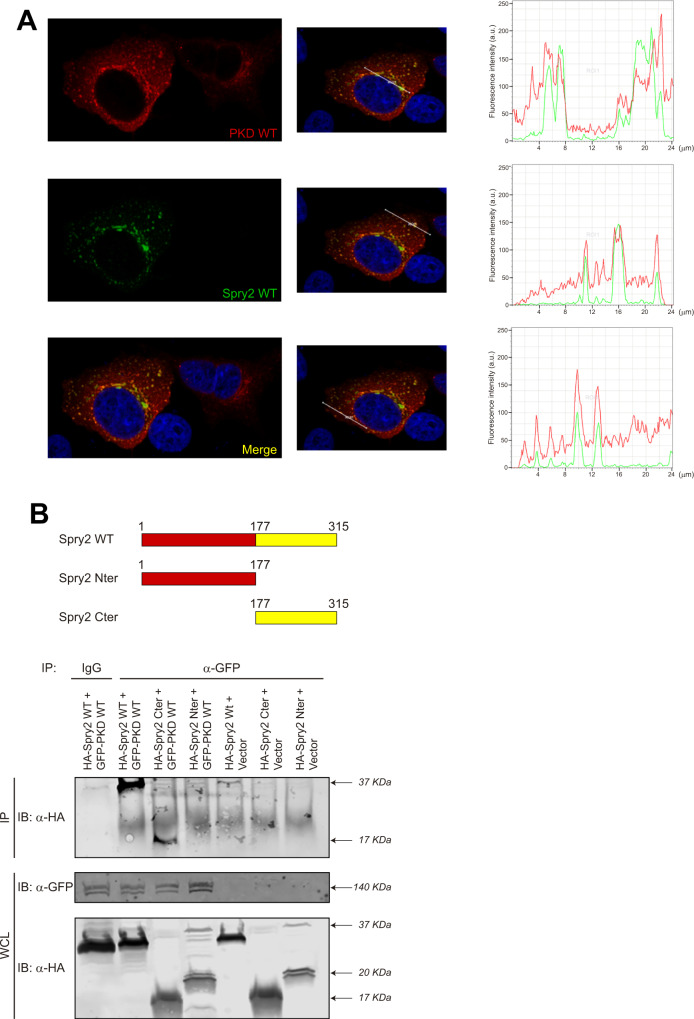
Spry2 associates with PKD through its C-terminal region. **A** PKD and Spry2 co-localize in vivo. HeLa cells transiently co-transfected with pECFL-AU5-Spry2 WT and pcDNA3-PKD1 WT were used for immunofluorescence-confocal microscopy analysis. Spry2 is visualized in green (by anti-AU5 FITC), PKD1 in red (by anti-PKD1 with a far-red-emitting fluorophore conjugate) and nuclei are stained with DAPI in blue. Panels show different localization and intensity profiles of PKD1 (red) and Spry2 (green), along different straight lines (white) of a representative cell. **B** The C-terminal region of Spry2 mediates its association with PKD in vivo. Cell extracts from transiently transfected HEK293T cells overexpressing Spry2 WT, Spry2 Cter (truncated mutant containing residues from 177 to 315) or Spry2 Nter (residues from 1 to 177), with HA epitope tag together with GFP-PKD WT or GFP vector alone, were incubated with anti-GFP polyclonal rabbit antibody or with unspecific rabbit IgG. Anti-GFP immunoprecipitates or WCL were then analyzed by immunoblotting using anti-GFP and anti-HA antibodies as described in Materials and Methods.

To ascertain whether Spry2 and PKD co-localize in the same cellular compartment we used immunofluorescence-confocal microscopy to analyze transiently co-transfected HeLa cells overexpressing both AU5-Spry2 WT and PKD1 WT. Our assays identified different areas of cellular co-localization of these proteins (Fig. 2A), further supporting in vivo association between Spry2 and PKD1 within these cells.

In addition, the region of Spry2 involved in association with PKD1 was further identified by co-immunoprecipitation assays. HEK 293 T cells were then transiently transfected with pEFBOS-GFP-PKD1 WT (GFP-PKD) or pEFBOS-GFP (Vector) together with pCEFL-KZ-HA-Spry2 WT (HA-Spry2 WT), or pCEFL-KZ-HA-Spry2-Nt (HA-Spry2-Nter: truncated mutant spanning residues 1–177 of Spry2), or pCEFL-KZ-HA-Spry2-Ct (HA-Spry2-Cter: truncated mutant spanning residues 177–315 of Spry2; Fig. 2B). The corresponding cytoplasmic extracts were incubated with anti-GFP polyclonal rabbit antibody or with unspecific rabbit IgG, and analyzed by immunoblotting using anti-HA monoclonal antibodies (Fig. 2B). These assays showed specific interaction of HA-Spry2 WT full-length protein with GFP-PKD: HA-Spry2 WT co-immunoprecipitates were only visualized in cells overexpressing HA-Spry2 WT + GFP-PKD WT (Fig. 2B), but not in cells carrying HA-Spry2 WT + Vector, nor in cell extracts incubated with nonspecific antibodies. Likewise, we found that, among the truncated Spry2 mutants, just HA-Spry2-Cter bound to GFP-PKD specifically, since this fragment was only co-immunoprecipitated from cells expressing HA-Spry2-Cter + GFP-PKD WT, but not from cells with HA-Spry2-Cter + Vector. By contrast, we did not detect any co-immunoprecipitation between the HA-Spry2-Nter fragment and GFP-PKD (Fig. 2B). All these observations demonstrate that Spry2 and PKD1 associate in vivo, and through the involvement of the carboxyl-terminal half of Spry2.

### Mutation of serine 112 by alanine or glutamic acid confers stability to Spry2

**Fig. 3:**
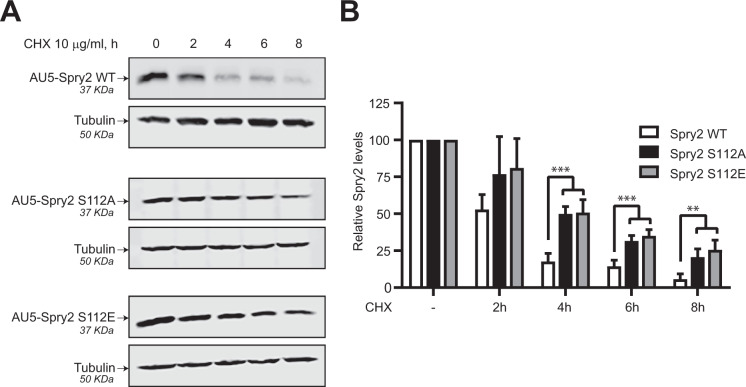
Spry2 S112A and S112E mutant proteins are more stable than Spry2 WT. **A** SW480-ADH cells constitutively overexpressing Spry2 WT, Spry2 S112A, or Spry2 S112E with AU5 epitope were treated with cycloheximide (CHX) 10 µg/ml for different times up to 8 h. AU5-Spry2 and tubulin levels were assessed by immunoblotting with specific antibodies after resolving WCL by SDS-PAGE. The images shown are representative of three independent experiments. **B** Quantitation of AU5-Spry2 protein levels normalized to tubulin levels. The histograms represent the average and SD of three separate assays (^**^p < 0.01, ^***^p < 0.001).

To investigate the long-term stability of cellular Spry2 protein and the impact of mutation at serine 112, SW480-ADH cells overexpressing ectopically AU5-Spry2 WT or mutants AU5-Spry2 S112A and S112E were incubated, in absence or presence of cycloheximide (CHX) for up to 8 h, and the decay of each protein was followed over time by immunoblotting using anti-AU5 antibody (Fig. 3A). Quantitation of the signals arising from the WT and mutant AU5-Spry2 samples showed that the substitution of serine at position 112 by alanine (no charge) or glutamic acid (negative charge) significantly increased the levels of the resulting cellular mutant protein (Fig. 3B). The fact that both the Spry2 S112A and Spry2 S112E mutants were more stable than the WT version of Spry2 suggests that this effect does not depend on the difference in electrical charge due to these two amino acids, but might be related to the phosphorylation state of Spry2 WT at serine 112. In addition, glutamic acid negative charge does not seem to be able to mimic the phosphorylation state of serine 112.

### PKD activity decreases the stability of WT Spry2 protein but not of Spry2 S112A mutant

**Fig. 4:**
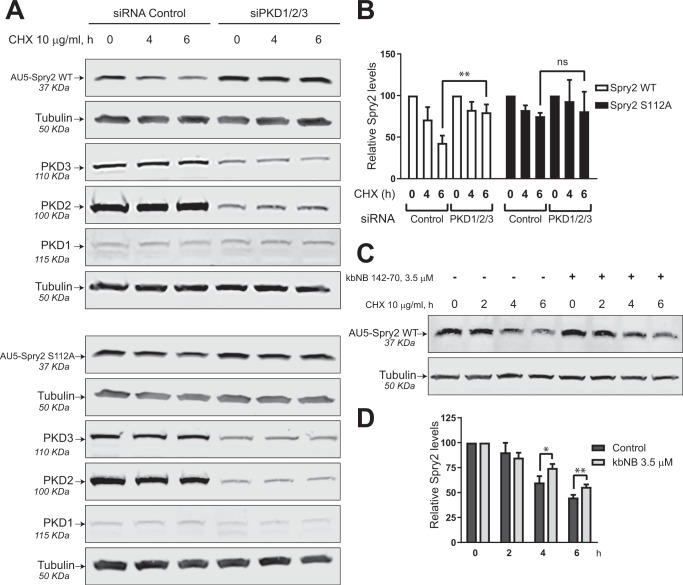
PKD negatively affects the stability of Spry2 WT but not of mutant Spry2 S112A. **A** SW480-ADH cells constitutively overexpressing AU5-Spry2 WT or AU5-Spry2 S112A were transfected with 37.5 nM control siRNA scramble (siRNA control), or 5 nM siRNAs PKD1 and 20 nM siRNAs PKD2 and 12.5 nM siRNAs PKD3 for 48 h. Cells were then treated with CHX (10 µg/ml) for 0, 4, and 6 h. Levels of AU5-Spry2 WT, AU5-Spry2 S112A, PKD1, PKD2, PKD3, and tubulin were detected by immunoblotting with specific antibodies after WCL were resolved by SDS-PAGE. The data shown are representative of three separate experiments. **B** Quantitation of AU5-Spry2 protein levels normalized to tubulin levels. Histograms represent the average and SD of three separate analysis (ns not significance, ^**^p < 0.01). **C** SW480-ADH cells constitutively overexpressing AU5-Spry2 WT were treated with CHX (10 µg/ml) for 0, 2, 4, and 6 h ±PKD-inhibitor 3.5 µM kbNB 142-70. **D** Quantitation of AU5-Spry2 levels normalized to tubulin levels. Histograms represent the average and SD of three separate assays (^*^p < 0.05, ^**^p < 0.01).

To examine whether Spry2 protein stability/homeostasis is affected by PKD, we knocked-down all three PKD isoforms using specific siRNAs in SW480-ADH cells ectopically overexpressing AU5-Spry2 WT or AU5-Spry2 S112A and then tested the level of AU5-Spry2 protein by immunoblotting using specific antibodies (Fig. 4A). Consistent with our previous qRT-PCR analyses (data not shown) of the expression levels of the three members of the PKD family, we detected high levels of PKD2 expression, intermediate levels of PKD3, and very low PKD1 expression (Fig. 4A). Notice that for these experiments we chose to use siRNAs targeting all three PKD isoforms to prevent any potential rebound/feedback overexpression of any isoform as consequence of reduced levels of the others. All three isoforms were effectively silenced under our experimental conditions (Fig. 4A). Cells transfected with control scrambled siRNA or with the pool of PKD-specific siRNAs were incubated in the absence or presence of cycloheximide (CHX) for 4 h and 6 h and levels of AU5-Spry2 protein were then quantitated and compared over time by immunoblotting with anti-AU5 antibody. We observed significantly higher levels of WT Spry2 protein after 6 h of CHX treatment in PKD1/2/3-silenced cells as compared to their corresponding controls transfected with scrambled siRNA (Fig. 4A, B). However, in sharp contrast, no differences were detected among the levels of mutant S112A-Spry2 protein when PKD isoforms were silenced in cell cultures overexpressing this mutant (Fig. 4A, B). These data strongly support the ability of PKD to reduce the intracellular stability of WT Spry2 protein through a mechanism involving, at least in part, the phosphorylation at serine 112. In order to confirm this notion, SW480-ADH cells constitutively overexpressing ectopic WT AU5-Spry2 protein were treated with cycloheximide for 0, 2, 4, and 6 h with or without the addition of the PKD-inhibitor kbNB 142-70 (Fig. 4C). The increased levels of AU5-Spry2 WT protein in the presence of PKD-inhibitor were statistically significant after 4 h and 6 h of treatment with CHX (Fig. 4D), suggesting that Spry2 protein stability is compromised upon PKD phosphorylation. Furthermore, no changes were detected in AU5-Spry2 S112A mutant protein levels regardless of PKD expression status, supporting the mechanistic relevance of the phosphorylation at serine 112 of Spry2 by PKD in modulation of the half-life of this protein (Fig. 4B).

### CSN3 knockdown increases WT Spry2 stability without altering Spry2 S112A mutant

PKD is described to associate with the COP9/Signalosome complex by interacting with the COP9 subunit CSN3 [20]. The COP9/Signalosome (CSN) complex is a conserved protein complex carrying isopeptidase activity that controls eukaryotic protein stability through the UPS (Ubiquitin-Proteasome system) by regulating the functionality of the cullin-RING ligase (CRL) families of ubiquitin E3 complexes [17].

**Fig. 5:**
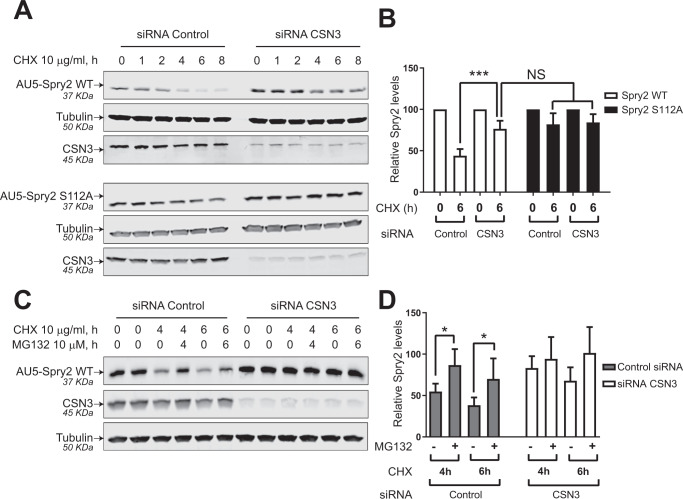
CSN3 negatively regulates the stability of Spry2 WT. **A** SW480-ADH cells, constitutively overexpressing AU5-Spry2 WT or AU5-Spry2 S112A, were transfected with either 60 nM control siRNA scramble (siRNA control), or a pool of four specific CSN3 siRNA (siRNA CSN3) for 48 h. Cells were then treated with CHX (10 µg/ml) up to 8 h. Levels of AU5-Spry2 proteins, CSN3 and tubulin were detected by immunoblotting with specific antibodies after whole-cell lysates were resolved by SDS-PAGE. Images shown are representative of three independent experiments. **B** Quantitation of AU5-Spry2 levels normalized to tubulin levels. Histograms represent the average and SD of three separate assays (ns not significance, ^***^p < 0.001). **C** SW480-ADH cells constitutively overexpressing AU5-Spry2 WT were transfected with either 60 nM control siRNA scramble (siRNA control), or specific CSN3 siRNAs (siRNA CSN3). 48 h after the transfection the cells were treated as indicated with CHX (10 µg/ml) and/or MG132 (10 µM) up to 6 h. Levels of Spry2, CSN3, and tubulin were detected by immunoblotting with specific antibodies after WCL were resolved by SDS-PAGE. The result shown is representative of three separate assays. **D** Quantitation of AU5-Spry2 levels normalized to tubulin levels. Histograms represent the average and SD of three separate assays (^*^p < 0.05).

In order to discern whether Spry2 protein stability could be modulated by the CSN complex, we knocked-down the CSN3 expression (using specific siRNAs) in SW480-ADH cells that ectopically overexpress AU5-Spry2 WT or the mutant AU5-Spry2 S112A (Fig. 5). Transfection of these cell lines with a pool of four specific siRNAs covering a portion of the human CSN3 coding sequence resulted in significant decrease of endogenous CSN3 expression as compared to the cells transfected with control, scrambled siRNA (Fig. 5A). To investigate the long-term stability of Spry2, the same cell lines were incubated in absence or presence of cycloheximide (CHX) for up to 8 h, and the decay of the AU5-Spry2 proteins (WT and S112A mutant) was monitored over time by immunoblotting using an anti-AU5 antibody (Fig. 5A). Quantitation of AU5-Spry2 levels normalized to those of tubulin showed that CSN3 silencing was associated with a significant stabilization and maintenance over time of the intracellular content of AU5-Spry2 WT protein (Fig. 5B). However, as expected, levels of AU5-Spry2 S112A mutant protein remained essentially unchanged upon CSN3 silencing (Fig. 5B). These results indicate that the half-life of Spry2 WT protein increases significantly after CSN3 knockdown and suggest that the CSN complex is also involved in regulating the Spry2 proteins intracellular stability. The above results are also consistent with the relevance of serine 112 for Spry2 protein durability, in concordance with the observed effects of Spry2 phosphorylation by PKD.

Since the CSN complex is able to control protein degradation through the Ubiquitin-Proteasome system (UPS) [21], we next decided to assess whether CSN3-dependent destabilization of Spry2 could be mechanistically related to Spry2 protein processing by the 26S proteasome pathway. To this end, SW480-ADH cells constitutively overexpressing AU5-Spry2 WT, with CSN3 expression knocked-down (using specific siRNAs), were treated with the proteasome inhibitor MG132 together with CHX for 4–6 h and then Spry2 protein levels were checked by immunoblotting (Fig. 5C). Quantitative analysis of the immunoblot signal indicated that MG132 treatment for 4–6 h, protected Spry2 protein from further degradation in the presence of CHX (Fig. 5D). Furthermore, when CSN3 expression was reduced by specific siRNAs, the relative Spry2 protein levels were stabilized independently of the 26S proteasome functionality (Fig. 5C, D).

## Discussion

Spry2 is a regulator of different RTK signaling pathways that has been postulated to modulate the stability (half-life) of a variety of pivotal proteins within those pathways [22]. Thus, Spry2 has been described to act as a scaffold protein bringing different ubiquitin E3 ligases close to their ubiquitination and degradation substrates, such as HIF1α (ubiquitinated by pVHL/associated E3 ligase) [23], the FRS2/FGFR complex and the β-subunit of the interleukin-6 receptor (both ubiquitinated by c-Cbl upon ligand stimulation) [22]. In contrast, binding of Spry2 to c-Cbl and the endocytic protein CIN85 [11] results in sustained EGFR stability [10]. Likewise, Spry2 has also been shown to bind to c-MET blocking its degradation, whereas Spry2 knockdown provokes the c-MET protein degradation [24]. This variant Spry2 behavior, linked to specific stimuli or cell types, seems to depend on its binding to protein phosphatase 2 A (PP2A) [22] and the resulting dephosphorylation of certain residues of the serine-rich motif of Spry2 (in particular serine 115 and 118) [22]. All four mammalian Spry proteins show a strong conservation and high number of residues (mainly serine and some threonine). In particular, the serine-rich motif of Spry2 spanning from position 107 to 132 contains consensus phosphorylation sites for several serine/threonine kinases (such as CK1/2, GSK3, Mnk1/2, and TESK1) [25]. It has been hypothesized that this conserved serine motif could be part of a key hinge region in mammalian Spry proteins, so that dephosphorylation within the motif (for instance, serine 115 and serine 118 of Spry2) would generate the “on” position of the switch and phosphorylation would correspond to the “off” state [22] thus controlling the intracellular stability of the Spry2 protein. As well as Spry2 exerts its effects by direct interaction with other proteins, its own levels can also be affected [22]. Indeed, the regulation of Spry2 protein stability is a complex process, involving the possibility of being poly-ubiquitinated by different E3 ubiquitin ligases for its degradation in the proteasome, such as Cbl [26], Siah2 [27], Nedd4 [25], or pVHL/associated E3 ligase [28].

In a search for cellular protein kinases able to phosphorylate Spry2, here we found that its serine-rich motif contains a consensus phosphorylation sequence for PKD (-(L/V/I)*X*(R/K)*XX*(S/T)-[18], “LSRSI**S**^112^”), which also appears in Spry1 (LSRST**S**^112^) and only partially in Spry3 (LPQHL**S**^80^), but is absent in Spry4. To confirm the functional relevance of this phosphorylation sequence, we demonstrate here that constitutively active PKD is able to phosphorylate WT Spry2 (in vitro and in vivo), but not the mutant protein at serine 112 (Spry2 S112A). In addition, using both confocal microscopy analysis and co-immunoprecipitation assays we have shown that Spry2 and PKD are associated in vivo through the C-terminal half of Spry2. It is worth noticing that serine 112 of Spry2 is also phosphorylated by other kinases besides PKD, such as Mnk1/2 [25] or p38^α^ (Martínez N et al., unpublished results), constituting a phosphorylation hotspot but however, only PKD is able to co-localize and co-immunoprecipitate with Spry2. It was previously described that Spry2 can form a ternary complex with PKCδ and PKD [29]. Although we cannot rule out that such a ternary complex is formed under our experimental conditions, the interaction with PKCδ requires the full-length Spry2 protein [29], whereas a truncated Spry2 mutant lacking the N-terminal half is able to form complexes with PKD1. Our data show that PKD negatively affects the half-life of the Spry2 protein, probably due to its kinase activity. Thus, both downregulation of the endogenous levels of PKD isoforms (using specific siRNAs), as well as treatment of cells with the PKD-inhibitor kbNB 142-70, significantly increased Spry2 WT protein lifetime. Consistently, we also found that the Spry2 S112A mutant protein was significantly more stable than the Spry2 WT version and its durability was not affected after silencing all PKD isoforms. Furthermore, we have also shown that both the S112A and S112E mutant Spry2 proteins are more stable than the WT Spry2, indicating that this effect does not depend on the differences in the electrical charge due to each changed residue, but could likely be related to the phosphorylation state of serine 112 in Spry2 WT.

It was previously reported that Mnk1 phosphorylates serines 112 and 121 of Spry2 [15], leading to increased stability of the Spry2 protein as compared to the non-phosphorylated form. Nevertheless, a subsequent report [25] analyzing the knockdown of endogenous Mnk1 and Mnk2, has proven that the latter isoform, but not Mnk1, is responsible for this dual phosphorylation and, more importantly, that phosphorylation of both serines (112 and 121) is essential for the interaction between Spry2 and Nedd4 (a member of the HECT-domain family E3 ubiquitin-ligase), leading to poly-ubiquitination and decreased stability of Spry2 [25]. PKD isoforms are known to be associated to the COP9-signalosome (CSN) complex [20] via molecular coupling with the CSN3 subunit of COP9 [20] and here we have demonstrated that the Spry2 protein stability was modulated by CSN.

CSN is a highly conserved multiprotein complex that regulates the Ubiquitin-Proteasome system (UPS) pathway in eukaryotes [17]. It encompasses nine subunits (CSN1–9), each one essential for the functionality of the complex and is also produced independently prior to coordinated assembly [17]. In particular, the function of the CSN3 subunit resides in stabilization of the CSN complex [17]. The main role of CSN is to regulate the cullin-RING ligase family of ubiquitin E3 complexes (CRLs) by eliminating Nedd8 from cullins [30]. In addition, CSN also acts as a scaffold platform that recruits several serine/threonine protein kinases (as CK2, AKT, and PKD) to regulate ubiquitination through E3 ubiquitin ligases of the UPS pathway, thus affecting the stability of different cellular proteins [17]. Indeed, PKD binds to CSN3 and modifies the CSN7 subunit [20]. Furthermore, the CSN-associated PKD phosphorylates several signaling proteins that are substrates of the UPS pathway, including c-Jun, p53 [20] or Sos1 [31], with different functional consequences. Specifically, CSN-mediated phosphorylation of p53 leads to its degradation [20], whereas this CSN-associated kinase activity stabilizes c-Jun [20] and Sos1 proteins [31]. Regarding Spry2 and in agreement with the results of reduction of PKD isoform levels, here we found that the stability of the Spry2 WT protein was significantly increased after CSN3 depletion, whereas the half-life of the Spry2 S112A mutant protein was unaffected by CSN3 knockdown. Furthermore, treatment with the 26S proteasome inhibitor MG132 has protected Spry2 WT protein from degradation, indicating that Spry2 destabilization is mechanistically related to its processing through the UPS pathway. However, the levels of Spry2 WT protein stabilized by CSN depletion were unaffected by the aforementioned proteasome inhibitor, supporting a role for CSN in controlling Spry2 protein degradation through the UPS pathway. Taken together, all these observations strongly indicate that the CSN/PKD complex is involved in control of the intracellular homeostasis of the Spry2 protein, although a full unraveling of the specific mechanistic details responsible for this effect remain to be clarified in future research.

Interestingly, our recent studies document that two proteins involved in the regulation of the RAS-RAF-MEK-ERK signaling pathway, Sos1 (a GEF activator of RAS proteins) and Spry2, appear to undergo opposite modulation of their molecular levels by the CSN/PKD complex. Thus, whereas Sos1 protein is stabilized by CSN/PKD action [31], the CSN/PKD complex induces Spry2 degradation (this report). Overall, our data are consistent with the oncogenic role that has been proposed for both the CSN3 protein and CSN as a whole [32], and support the consideration of the CSN/PKD complex as a relevant potential target in the quest for new therapeutic drugs.

## Materials and methods

### Cell lines, transfections

Human colon carcinoma SW480-ADH, HEK293T embryonal kidney and HeLa cell lines were cultured in Dulbecco’s modified Eagle’s medium (DMEM; Invitrogen, Paisley, United Kingdom) supplemented with 10% fetal calf serum (FCS; Invitrogen). SPRY2-wt, SPRY2-S112A, and SPRY2-S112E and Mock cells were generated by stable transfection of SW480-ADH cells with pCEFL-KZ-AU5, pCEFL-KZ-AU5-SPRY2 WT, pCEFL-KZ-AU5-SPRY2 S112A, or pCEFL-KZ-AU5-hSPRY2 S112E plasmids, as described previously [6]. Cell lines were authenticated by the Genomics Service of Instituto de Investigaciones Biomédicas Alberto Sols (IIBM, CSIC-UAM, Madrid, Spain) using the *GenePrint*® 10 System of Promega (Madison, WI, USA), which allows co-amplification and three-color detection of ten human *loci*: TH01, TPOX, vWA, Amelogenin, CSF1PO, D16S539, D7S820, D13S317, D21S11, and D5S818. These *loci* together provide a genetic profile with a random match probability of 1 in 2.92 × 10^9^ and are used for human cell line and tissue authentication and identification and human cell line cross-contamination determination. STRs profiles were compared against cell line databases like ATCC (American Type Culture Collection) and DSMZ (Deutsche Sammlung von Mikrorganismen and Zellkulturen). Cells were tested routinely to ensure there was no mycoplasma contamination (Universal Mycoplasma Detection kit, ATCC, Manassas, VA, USA; #30–1012). Transient transfections of HEK293T cells were performed in p100 plates using Jet-Pei (Polyplus-Transfection, Illkirch, France). Twenty-four hours post-transfection, cells were serum-starved by incubation in DMEM supplemented with 0.5% FBS for 18 h. All assays were done 48 h after transfection.

### Antibodies and reagents

Monoclonal mouse antibodies against CSN3 and PKD1 were purchased from Santa Cruz Biotechnology (Santa Cruz, CA). Anti-HA and anti-AU5 monoclonal antibodies from Berkeley Antibody Company (Berkeley, CA); polyclonal rabbit antibodies against PKD2 and PKD3 from Cell Signaling Technology (Boston, MA, USA); polyclonal rabbit antibody against CSN3 was acquired from Abcam (Ab79698-Cambridge, UK); polyclonal rabbit anti-GFP rabbit A6455 from Invitrogen and anti-beta-tubulin monoclonal antibody T4026 was acquired from Sigma–Aldrich (St Louis, MO, USA). The bound antibodies were detected in some experiments with secondary antibodies conjugated with IRDye680 or IRDye800 and analyzed with an Odyssey Imager system (LI-COR, Lincoln, NE, USA), or anti-mouse or anti-rabbit horseradish peroxidase (1:5000; Bio-Rad, Hercules, CA, USA) secondary antibodies for visualization using an Enhanced Chemiluminescence Detection Kit (Amersham, Arlington Heights, IL, USA). Cycloheximide (C1988) and MG132 (M7449) were purchased from Sigma–Aldrich (St. Louis, MO, USA) and the PKD-inhibitor kbNB 142-70 was obtained from Bio-Techne Corporation (Minneapolis, MN, USA). The siRNAs siGENOME SMART pool human Cops3 (M-011494-00), siGENOME SMART pool human Prkd1 (M-005028-02), siGENOME SMART pool human Prkd2 (M-004197-02), siGENOME SMART pool human Prkd3 (M-005029-01), and siGENOME Control pool (D-001206-14) were purchased from Dharmacon (Lafayette, CO, USA). Carrier-free Phosphorus-32 radionuclide (314-327TBq/mMole) and [γ^32^P]-ATP (370 MBq/ml) were purchased from PerkinElmer, Inc. (Boston, MA, USA). Goat anti-Rabbit IgG (H + L) Secondary Antibody, Texas Red-X (T6391) was purchased from Thermo-Fisher Scientific, and FITC-conjugated anti-AU5 antibody from Covance.

### DNA constructs

Full-length cDNA of human Spry2 was obtained by RT-PCR from total RNA of Molt-4 cells using primers designed for the published sequence and providing *Bgl*II and *Not*I sites at the 5′ and 3′ ends, respectively [33]. The amplified product was then subcloned into *Bgl*II and *Not*I sites of pCEFL-KZ-AU5 as described previously [6]. The mutant Spry2 S112A and Spry2 S112E was generated from the above pCEFL-KZ-AU5-Spry2 wild-type (WT) by site-directed PCR-mutagenesis, using specific primers in which serine was substituted for alanine as described previously [6], or glutamic acid, respectively, and subcloned into *Bgl*II and *Not*I sites of pCEFL-KZ-AU5. The N-terminal (Nter-hSpry2) and C-terminal (Cter-hSpry2) portions of Spry2 WT (coding regions 1–531, and 532–945, respectively) were cloned as described previously [33]. The vectors pcDNA3-PKD1 WT, pEFBOS-GFP, pEFBOS-GFP-PKD1 WT, pEFBOS-GFP-PKD1-CA (GFP-PKD-CA), and pEFBOS-GFP-PKD1-KD (GFP-PKD-KD) were used for the ectopic expression of PKD1 and different mutants that had been generated previously [19, 34].

### Immunoprecipitation and immunoblotting

The cells were lysed in buffer containing HEPES 25 mM pH 7.5, 150 nM NaCl, 1% NP40, protease inhibitor cocktail (catalog number P8340) from Sigma–Aldrich and phosphatase inhibitor cocktail (HY-K0022) from MCE. Nucleus-free supernatants were incubated with appropriate antibodies as described [33]. Immune-complexes and cell extracts were resolved by SDS-PAGE, before being transferred to nitrocellulose (Immobilon P, Millipore) membranes and visualized by Odyssey Imager system using appropriate antibodies.

### RNA interference

In knockdown assays, siRNA duplexes were transfected twice in SW480 cells using Lipofectamine RNAiMAX Reagent (Invitrogen) at 24 h intervals. To knockdown CSN3 and PKD1/2/3 was used siGENOME SMART pool human Cops3 (M-011494-00), siGENOME SMART pool human Prkd1 (M-005028-02), siGENOME SMART pool human Prkd2 (M-004197-02), siGENOME SMART pool human Prkd3 (M-005029-01), respectively, and for siRNA control siGENOME Control pool (D-001206-14).

### In vitro kinase assays

In vitro kinase assays were performed as described [19] by immunoprecipitated kinase (GFP-PKD-KD or GFP-PKD-CA) using anti-GFP antibodies with AU5-Spry2 immunoprecipitates in the presence of [γ-^32^P] ATP. Immunoprecipitates were analyzed by SDS-PAGE and autoradiography.

### In vivo labeling

HEK293T cells transiently transfected with GFP-PKD and AU5-Spry2 WT or AU5-Spry2 S112A for 3 days were serum deprived for 2 h, incubated at 37 °C in phosphate-free DMEM for 4 h, and then metabolically labeled with the same medium containing 200 μCi/ml carrier-free ^32^P_i_ for 5 h as we performed previously [19]. At the end of this labeling period, cells were lysed and immunoprecipitated with anti-AU5-antibody and analyzed by SDS-PAGE and autoradiography.

### Confocal microscopy

Transfected cells grown on coverslips were fixed for 15 min with 4% paraformaldehyde in phosphate-buffered saline at 4 °C. After blocking (5% fetal bovine serum in phosphate-buffered saline for 30 min), cells were incubated with the corresponding primary antibodies for 1 h at room temperature, and immunoreactivity was detected with the suitable fluorophore-conjugated secondary antibody before mounting on slides with Mowiol 4–88 (Harland, Co, UK). All confocal images were acquired using an inverted Leica laser confocal microscope SP5 with a ×63 Plan-Apochromatic oil immersion objective and were normalized for each color separately.

### Statistical analysis

Data were assessed by the GraphPad Prism 9.3.1 (San Diego, CA, USA). Results were expressed as mean ± SD of the indicated number of experiments. Normality of the data was evaluated and statistical significance was assessed using the *t*-test for unpaired observations. Immunoblotting results were analyzed using linear correlations between increasing amounts of protein and signal intensity.

## Data Availability

All data generated or analyzed during this study are included in this published article.
